# Laser acupuncture improves post-dialysis fatigue and sleep quality independent of time-varying dialysis and depression symptoms: a double-blind, randomized, placebo-controlled trial

**DOI:** 10.1186/s12906-025-05203-3

**Published:** 2025-12-13

**Authors:** Rou-Yu Sung, Mei-Ling Yeh, Fang-Pey Chen, Ming Yu Lo, Jeannie Yu

**Affiliations:** 1 Department of Nursing, Taipei Veterans General Hospital, School of Nursing, National Taipei University of Nursing and Health Sciences, Taipei, Taiwan; 2 School of Nursing, National Taipei University of Nursing and Health Sciences, Taipei, Taiwan; 3 School of Chinese Medicine, College of Medicine, National Yang Ming Chiao Tung University; Center for Traditional Medicine, Taipei Veterans General Hospital, Taipei, Taiwan; 4 School of Chinese Medicine for Post Baccalaureate, I-Shou University, Kaohsiung, Taiwan

**Keywords:** End-stage renal disease (ESRD), Depression, Fatigue, Hemodialysis (HD), Laser acupuncture, Sleep quality

## Abstract

**Background:**

Patients with end-stage renal disease (ESRD) who undergo long-term hemodialysis (HD) often suffer from fatigue and poor sleep quality. Although acupuncture is known to help regulate and enhance bodily functions, it remains uncertain whether laser acupuncture can benefit these patients. This double-blind, randomized, placebo-controlled trial aimed to evaluate the effects of laser acupuncture on reducing fatigue and improving sleep quality in hemodialysis patients. The study also considered time-varying dialysis and depressive symptoms.

**Methods:**

Seventy-two patients were randomly assigned to either the laser acupuncture (LA) group or the control group. The LA group received acupuncture with a low-level laser at specific points: Hegu (LI4), Shenmen (HT7), Neiguan (PC6), Taixi (KI3), Zusanli (ST36), Yanglingquan (GB34), and Sanyinjiao (SP6). The control group received the same treatment without laser energy. Measurements were taken at baseline, Week 2, Week 4, and Week 6.

**Results:**

Adjusted for HD and depression symptoms, the mixed linear model showed significant improvements in the LA group compared to the control group and baseline scores. Notable improvements were observed in fatigue visual analog scale scores, HD-related fatigue scale scores, recovery time after HD, and Pittsburgh sleep quality index at Week 2, Week 4, and Week 6 (*p* < 0.05).

**Conclusions:**

A six-week laser acupuncture treatment significantly improved fatigue and sleep quality in patients with ESRD on HD, regardless of dialysis timing and depressive symptoms. The treatment is non-invasive, safe, and short, making it a promising option for the post-dialysis period. Future research should evaluate the long-term effects of laser acupuncture on patients with HD, and involving more medical centers could strengthen the significance of this study’s results.

**Implication for the profession and/or patient care:**

These findings provide valuable insights into the benefits of laser acupuncture for alleviating post-dialysis fatigue, shortening fatigue recovery time, and improving sleep quality in patients with ESRD undergoing HD. Starting in the second hour of the HD procedure, laser acupuncture can be applied to seven specific acupoints: LI4, HT7, PC6, KI3, ST36, GB34, and SP6. For the best results, it is recommended that laser acupuncture be performed for at least two weeks.

**Trial registration:**

This clinical trial was registered in ClinicalTrials.gov with registration NCT06028685 (31/07/2023).

**Supplementary Information:**

The online version contains supplementary material available at 10.1186/s12906-025-05203-3.

## Introduction

It is important to note that the number of end-stage renal disease (ESRD) cases has increased significantly in recent years, greatly raising the disease burden and posing unprecedented challenges for healthcare worldwide. According to the latest report by the United States Renal Data System, Taiwan has the highest prevalence of ESRD (3,806 cases per million population) and the highest incidence of treated ESRD (536 cases per million people) globally as of 2022. Hemodialysis (HD) remains a crucial treatment for ESRD patients [1]. Hemodialysis (HD) is a life-sustaining treatment for ESRD patients. However, these patients undergoing long-term hemodialysis often face multiple complications, such as fatigue [2], insomnia [3], anemia [4], seizures [5], and depression [6].

Post-dialysis fatigue describes a state of extreme tiredness, weakness, and low energy that occurs after the dialysis procedure, significantly affecting patients' daily activities and requiring rest or sleep [7]. A systematic review found that the prevalence of post-dialysis fatigue can be as high as 61.0% [8]. The severity, duration, and frequency of this fatigue are associated with the intensity of dialysis symptoms [9]. Several factors contribute to post-dialysis fatigue, such as acidosis [10] and protein wasting [11]. Additionally, a systematic review identified a significant correlation between post-dialysis fatigue and sleep quality [12]. Sleep quality is a subjective measure of sleep characteristics such as depth, restfulness, time to fall asleep, duration, and the frequency of awakenings [13]. HD patients often experience sleep disturbances caused by physiological factors such as uremia [14], decreased melatonin levels [15], and lower vitamin D levels [16]. Increased dialysis-related symptoms can lead to anxiety, despair, and depression, all of which negatively impact sleep quality [17]. Poor sleep quality, in turn, worsens patients' fatigue [18].

Acupuncture-related treatments have been shown to improve fatigue and sleep quality in HD patients. One systematic review indicated that transcutaneous electrical acupoint stimulation, acupressure, and massage may improve fatigue in patients receiving dialysis, although the evidence is of low certainty [19]. Another review suggested that acupressure significantly enhances sleep quality in patients undergoing HD [20]. Similar to acupuncture, laser acupuncture is promising for improving sleep problems in individuals diagnosed with chronic insomnia [21]. Laser acupuncture is a form of alternative therapy that uses low-level lasers instead of needles to stimulate acupoints. It produces therapeutic effects through photobiomodulation [22]. Low-level laser therapy enhances local microcirculation, supports oxygen delivery to hypoxic cells, and aids in removing waste products from cell metabolism [23]. A review stated that the benefits of laser acupuncture include being non-invasive, painless, safe, and free of fear, with a short treatment duration [24]. Promoting adenosine triphosphate can regulate various physiological processes, boosting cellular energy and supporting metabolism [25]. In addition, the optimal dose applied directly to the target area is more important than the wavelength [23]. This study used lower doses and increased application frequency to treat fatigue. However, there is limited research on the effects of laser acupuncture in patients undergoing HD. To address this gap, this study aimed to evaluate the impact of laser acupuncture on reducing fatigue and improving sleep quality in HD patients. The study also considered variations in dialysis timing and depressive symptoms.

### Methods

#### Study design and participants

This double-blind, randomized, placebo-controlled trial was conducted at a 2,808-bed medical center in northern Taiwan and recruited patients aged 20 years and older who were diagnosed with ESRD, receiving thrice-weekly HD for at least three months, had clear consciousness for communication, and consented by signing the informed consent form. Patients with incomplete skin integrity or infective wounds at acupoint sites, those on immunosuppressants, photosensitivity, cardiac pacemakers, or sedative medications were excluded from the study. Participants were randomly assigned to the laser acupuncture (LA) group, which received laser acupuncture at specific acupoints for six weeks, or the control group, which received the same therapy without laser energy output. The effects were measured at baseline, Week 2, Week 4, and Week 6.

**Fig. 1:**
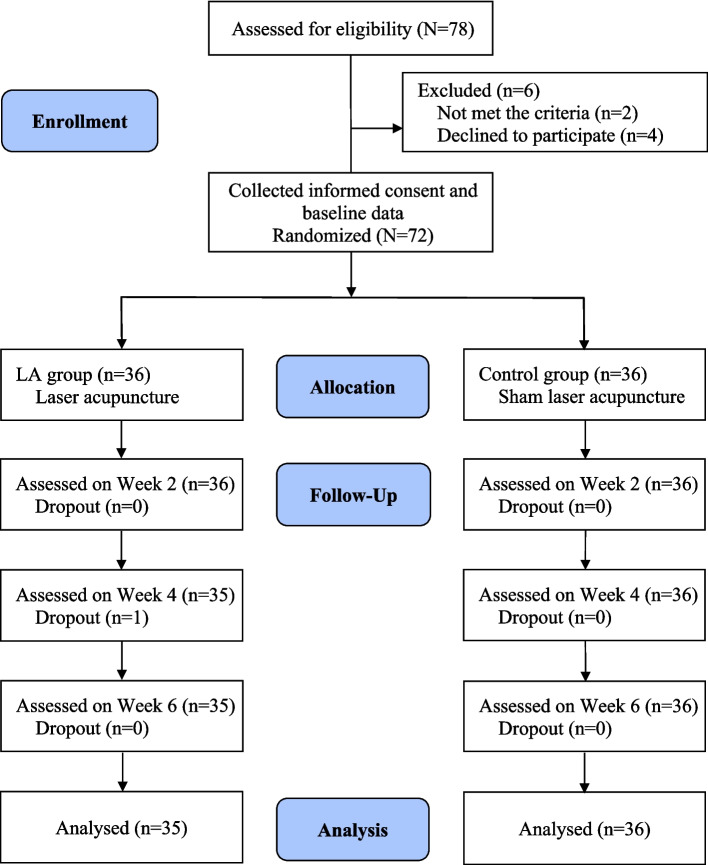
Flow chart of research design and participant allocation

Based on G-power 3.0 software analysis, using an effect size of 0.3 [26], an α error probability of 0.05, a power of 0.80, two groups, and four repetitions, the total sample size required was 58. To account for a 20% dropout rate, 72 participants were enrolled, with 36 in each group. The research team, who were not involved in the intervention or data collection, used a permuted block randomization procedure to allocate participants to groups. Random allocation sequences were generated by a computer and placed into sequentially numbered blocks of four in sealed, opaque envelopes. After obtaining informed consent and collecting baseline data, a researcher (RY) opened the envelopes according to the concealed sequence to assign participants to groups. Participants and their healthcare providers remained blinded to group assignments and interventions. Figure 1 shows a flowchart outlining the research design and participant allocation.

#### Intervention

**Fig. 2:**
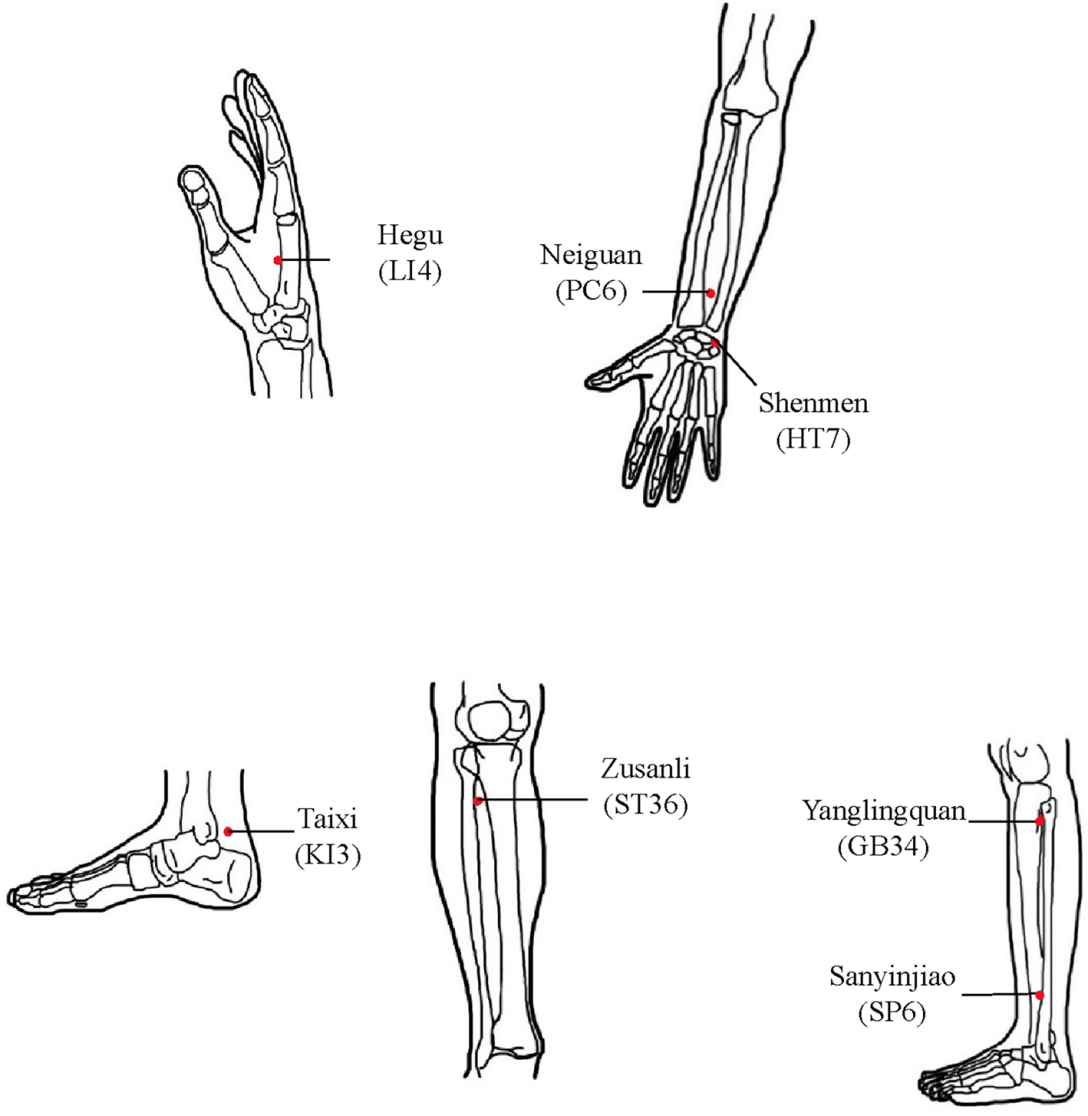
Location of acupuncture points for fatigue and sleep quality

The participants in the LA group received laser acupuncture during HD, starting from the second hour of the procedure. To avoid areas with arteriovenous fistulas, the medical team carefully treated the acupoints on the upper limbs. Seven acupoints were selected for intervention after a thorough review of the literature [20, 21, 27–29]. These included Hegu (LI4), Shenmen (HT7), Neiguan (PC6), Taixi (KI3), Zusanli (ST36), Yanglingquan (GB34), and Sanyinjiao (SP6), as shown in Fig. 2. Two systematic reviews concluded that LI4, HT7, PC6, KI3, ST36, and SP6 are commonly used for managing fatigue or insomnia [27, 28]. Specifically, ST36 and SP6, along with GB34, were applied to relieve fatigue in HD patients [29]. HT7 and SP6 target deficiencies in heart and spleen qi, while PC6 and KI3 address disharmony between heart and kidney qi; disruptions in these areas are associated with sleep disturbances [21]. Stimulating HT7 is viewed as a way to improve mental and emotional well-being, especially concerning insomnia and sleep disorders, making it a key acupoint [20].

The laser acupuncture treatment was conducted using the TRANS portable laser phototherapy device (T1-816-3E2, Taiwan, DOH Medical Device Manufacturing No.003912). This device emits a wavelength of 808 ± 10 nm, has an output power of 150 mW, and operates in continuous wave mode. It is of the Gallium-Arsenide-Aluminium (Ga-As-Al) type. Each acupoint was subjected to 20 s of low-level laser therapy, delivering a total dose of 3 J/cm^2^ (0.15 J × 20 s) output with direct contact and applied pressure. Systematic reviews suggest that the optimal energy density for laser acupuncture ranges from 1–4 J/cm^2^ [30] or 0.57–5 J per acupoint [31]. A total of seven acupoints were treated bilaterally. Participants in the control group received the same intervention as the laser acupuncture (LA) group, except that the placebo laser acupuncture targeted the acupoints without emitting any laser output. The subthermal, invisible infrared laser beam used in both active and placebo procedures was difficult for participants and clinicians to distinguish. Consequently, no noticeable physiological changes were observed.

Each treatment session lasted five minutes and was administered three times a week over a six-week intervention period, totaling 18 sessions. Before starting the study, participants' physiological parameters, such as blood pressure and blood oxygen saturation, were monitored using an electronic blood pressure monitor (Model VM4, Philips, United States). During laser acupuncture sessions, stimulation was immediately halted if participants experienced discomfort, such as sharp pain, dizziness, or nausea. Blood pressure and blood oxygen saturation levels were then re-evaluated. Prior to the study, the researcher (RY) obtained certification in laser acupuncture techniques from the Taiwan Traditional Chinese Medicine Association. Additionally, two attending physicians from medical centers confirmed the appropriateness of the acupoint selection and the correctness of the acupoint manipulation, achieving an expert validity test score of 100%.

#### Measurement instruments

This study used three scales to measure fatigue levels: the Fatigue Visual Analog Scale, the HD-related Fatigue Scale, and recovery time after HD. First, the Fatigue Visual Analog Scale was used to assess subjective fatigue levels. It features a 10 cm horizontal line, where 0 indicates no fatigue on the far left and 10 indicates complete exhaustion on the far right. Second, the HD-related Fatigue Scale assesses five main factors: reduction in vigor and motivation, reduction in physical ability, reduction in mental ability, reduction in daily activities, and distress and loss of mood control [32]. Each item is scored on a 4-point scale, from rarely or never happening (0) to often happening (3). Scores range from 26 to 104, with higher scores indicating more frequent fatigue. The Cronbach's α for internal consistency reliability of the HD-related Fatigue Scale was 0.93 [33], and it was 0.96 in this study. Finally, recovery time after HD was measured by asking dialysis patients how long it took them to recover from a session [34], which correlates well with post-dialysis fatigue (r = 0.508, *p* < 0.001) [35]. The correlation in this study was r = 0.441, *p* < 0.001.

The Pittsburgh Sleep Quality Index (PSQI) measures sleep quality over the past month and includes seven components: subjective sleep quality, sleep latency, sleep duration, habitual sleep efficiency, sleep disturbances, use of sleep medication, and daytime dysfunction [13]. Each item is rated from 0 to 3, resulting in a total score from 0 to 21, where higher scores signify poorer sleep quality. The Cronbach's α for internal consistency reliability of the PSQI ranged from 0.82 to 0.83 [36]; in this study, it was 0.76.

The Dialysis Symptom Index (DSI) includes 30 items, with 21 related to physical symptoms and nine related to emotional symptoms [37]. Symptom severity is rated on a 5-point Likert scale from not at all bothersome (0) to very bothersome (4). Scores range from 0 to 120, with higher scores indicating more severe physical and psychological symptoms. The Cronbach's α for internal consistency reliability of the DSI was reported as 0.90 [38]; in this study, it was 0.94. The Beck Depression Inventory (BDI) assesses behavior, emotion, cognition, and psychological aspects [39]. It consists of 21 items rated on a 4-point Likert scale from not at all or rarely (0) to often or always (3), with total scores ranging from 0 to 63. Normal scores are from 0 to 16, mild depression from 17 to 22, moderate depression from 23 to 30, and severe depression from 31 to 63. The Chinese version of the BDI was used in this study. The Cronbach's α for internal consistency reliability of the BDI was 0.92 [40]; in this study, it was 0.90.

All instruments were used to establish the baseline data. Additionally, the Fatigue Visual Analog Scale was administered to participants before and after the intervention. The other instruments were measured during Weeks 2, 4, and 6 of the intervention.

#### Statistical analysis

Data analysis was performed using IBM SPSS 26 software. Categorical variables were presented as sample sizes and percentages, while continuous variables were summarized as means and standard deviations (SD). A linear mixed model was used to assess the effect of laser acupuncture on fatigue and sleep quality, controlling for time-varying dialysis and depressive symptoms throughout the study. This statistical approach allowed for the examination of repeated measurements of the outcomes between the two groups. Statistical significance was set at *p* < 0.05. The data were analyzed by a statistician who was blinded to the participant groupings.

## Results

### Participant characteristics

Seventy-one participants completed the study, with a low dropout rate of 1.4%. One participant withdrew due to receiving a pancreas and kidney transplant in the LA group. No serious adverse events were reported by the research nurse; however, three participants experienced temporary, mild tingling sensations, and one had minor petechiae. The study included more female participants (54.3%) than male. The average age of participants was 59.69 years (SD 16.00) and 58.89 years (SD 10.35) for the LA and control groups, respectively. The mean dialysis duration for the LA and control groups was 7.80 years (SD 6.24) and 6.5 years (SD 4.37), respectively. Table 1 shows no significant differences in demographic and clinical characteristics between the two groups (*p* > 0.05).

**Table 1:** Demographic and baseline characteristics of participants

Variables	LA group (*n* = 35)		Control (*n* = 36)		χ^2^	*p*
	**n**	**%**	**n**	**%**		
Sex						
Female	19	54.3	16	44.4	0.35	0.56
Male	16	45.7	20	55.6		
Dialysis caregivers						
None	16	45.7	22	61.1	3.89	0.44
Family	17	48.6	12	33.3		
Homecare attendant	2	5.7	2	5.6		
Etiology of ESRD						
Diabetes	16	51.6	17	56.7		
Hypertension	18	58.1	20	66.7		
Cardiovascular disease	16	51.6	9	30.0		
Gout	1	3.2	1	3.3		
SLE	2	6.5	4	13.3		
Malignant tumor	3	9.7	2	6.7		
Medication						
Iron supplement	5	14.7	3	8.8		
Erythropoietin	33	97.1	33	97.1		
Nutrient injection	1	2.9	2	5.9		
Sleeping pills	2	5.9	1	2.9		
Antiepileptic drugs	3	8.8	0	0.0		
	**mean**	**SD**	**mean**	**SD**	**t**	***p***
Age (year)	59.69	16.00	58.89	10.35	0.25	0.81
Dialysis duration (hour)	3.84	0.27	3.85	0.24	−0.19	0.85
Dialysis experience (year)	7.80	6.24	6.50	4.37	1.02	0.31

Abbreviations: *LA group* laser acupuncture group, *ESRD* end-stage renal disease, *SLE* Systemic lupus erythematosus, *SD* standard deviation

### Main outcome measures

**Fig. 3:**
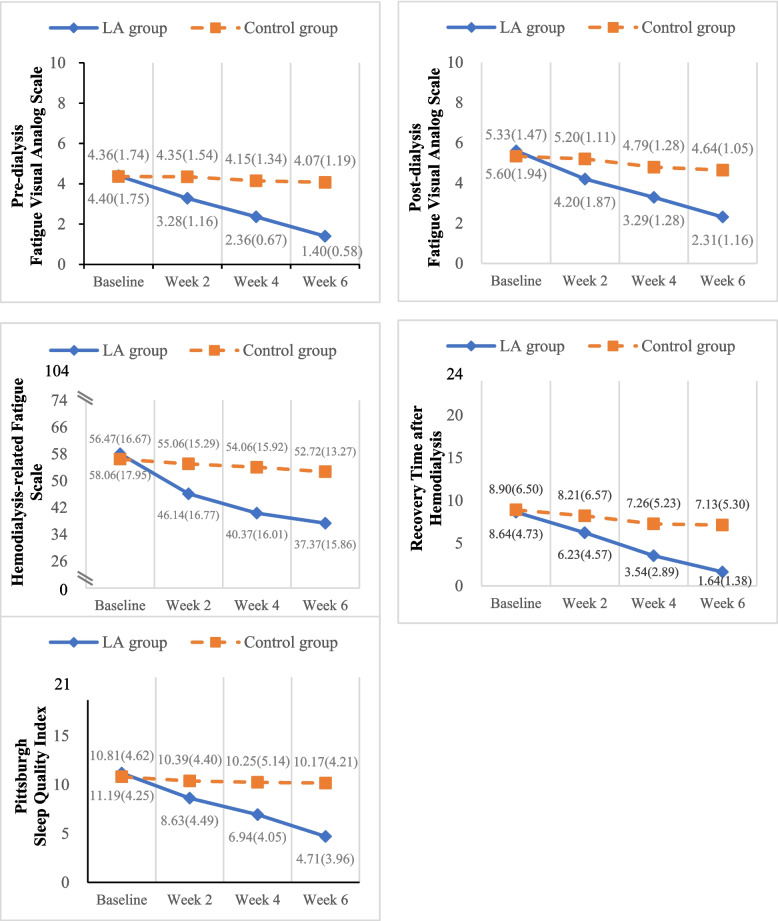
Trends of the outcomes of fatigue and sleep quality over time

The results showed changes in fatigue and sleep quality over time, with the LA group showing a better trend than the control group, as illustrated in Fig. 3. There were no significant differences at baseline between the two groups in the fatigue visual analog scale, HD-related fatigue scale, recovery time after HD, PSQI, DSI, and BDI, as shown in Table 2 (*p* > 0.05). After adjusting for time-varying DSI and BDI scores, the mixed linear model indicated significant differences in the LA group compared to the control group and baseline. These differences were particularly evident in the fatigue visual analog scale, HD-related fatigue scale, recovery time, and PSQI at Weeks 2, 4, and 6 (*p* < 0.05). DSI was associated with outcomes on the HD-related fatigue scale and PSQI, while BDI was linked with the HD-related fatigue scale.

**Table 2:** Result of the linear mixed model analysis for fatigue and sleep quality

Parameter	Intercept	LA group^a^	LA group*Week 2^b^	LA group*Week 4^b^	LA group*Week 6^b^	DSI	BDI
Pre-dialysis Fatigue Visual Analog Scale							
Estimate	4.05	0.02	−1.03	−1.76	−2.60	0.01	0.00
t	13.39	0.06	−4.04	−5.30	−6.86	1.38	0.43
*P*	< 0.001	0.95	< 0.001	< 0.001	< 0.001	0.17	0.67
95% CI	[3.45, 4.64]	[−0.61, 0.65]	[−1.54, −0.53]	[−2.41, −1.11]	[−3.34, −1.85]	[0.00, 0.02]	[−0.01, 0.02]
Post-dialysis Fatigue Visual Analog Scale							
Estimate	5.03	0.25	−1.21	−1.70	−2.48	0.01	0.00
t	16.06	0.73	−4.92	−5.24	−6.59	1.47	0.23
*P*	< 0.001	0.47	< 0.001	< 0.001	< 0.001	0.14	0.81
95% CI	[4.41, 5.65]	[−0.42, 0.91]	[−1.70, −0.73]	[−2.34, −1.06]	[−3.22, −1.74]	[0.00, 0.02]	[−0.02, 0.02]
Hemodialysis-related Fatigue Scale							
Estimate	33.02	0.59	−5.76	−9.06	−8.19	0.39	0.53
t	11.73	0.20	−2.25	−2.78	−2.24	8.44	5.81
*P*	< 0.001	0.84	0.03	0.01	0.03	< 0.001	< 0.001
95% CI	[27.47, 38.57]	[−5.14, 6.33]	[−10.81, −0.72]	[−15.48, −2.64]	[−15.41, −0.98]	[0.30, 0.48]	[0.35, 0.71]
Time of recovery after hemodialysis							
Estimate	8.02	−0.30	−1.48	−3.18	−4.81	0.00	0.05
t	7.79	−0.26	−2.06	−3.28	−4.22	0.10	1.59
*P*	< 0.001	0.79	0.04	< 0.001	< 0.001	0.92	0.11
95% CI	[5.99, 10.05]	[−2.58, 1.97]	[−2.90, −0.06]	[−5.09, −1.27]	[−7.06, −2.57]	[−0.03, 0.03]	[−0.01, 0.10]
Pittsburgh Sleep Quality Index							
Estimate	8.26	0.13	−1.68	−3.00	−4.83	0.08	−0.01
t	8.90	0.14	−2.06	−2.85	−4.06	5.10	−0.46
*P*	< 0.001	0.89	0.04	< 0.001	< 0.001	< 0.001	0.65
95% CI	[6.43, 10.09]	[−1.77, 2.04]	[−3.30, −0.07]	[−5.07, −0.92]	[−7.17, −2.49]	[0.05, 0.11]	[−0.07, 0.04]

*Covariate DSI* Dialysis Symptom Index, covariate *BDI* Beck Depression Inventory

^a^Reference: control group

^b^Reference: control group

*Baseline: interaction covariate

## Discussion

The findings of this study showed that laser acupuncture effectively reduced post-dialysis fatigue symptoms and shortened recovery time, while also improving sleep quality in ESRD patients undergoing HD. Their post-dialysis fatigue began to improve from the second week of the six-week treatment, and recovery time was reduced after laser acupuncture treatment. Post-dialysis fatigue was associated with both dialysis and depressive symptoms, whereas sleep quality was only associated with dialysis symptoms. However, the positive effects of laser acupuncture lasted for six consecutive weeks without any interference from various dialysis or depressive symptoms over time. This study is the first to demonstrate the effect of laser acupuncture in treating fatigue in ESRD patients undergoing HD. In this study, laser acupuncture was referred to as combined acupuncture with low-level laser therapy, producing effects similar to traditional acupuncture [22]. The findings of this study support a previous systematic review that found acupuncture effectively reduces cancer-related fatigue [27]. This also agree with a previous systematic review suggesting that acupressure and transcutaneous electrical acupoint stimulation may help improve dialysis-related fatigue [19]. Additionally, the improvement in sleep quality in this study was observed starting in the second week of intervention, with the effects gradually strengthening and lasting until the sixth week. This aligns with previous systematic reviews indicating that acupuncture effectively enhances sleep quality in patients with cancer-related insomnia [27, 28]. Furthermore, this study found that laser acupuncture could be administered during the second hour of HD, taking only 5 min for bilateral selected acupoints per session, which greatly reduces the intervention time [24].

The seven acupoints—LI4, HT7, PC6, KI3, ST36, GB34, and SP6—used in this study, which showed effects on fatigue recovery and sleep improvement, align with previous studies [20, 21, 27–29]. In targeting HD patients to enhance fatigue recovery, the application of LI4 and ST36 has been demonstrated [41, 42]. This also corresponds with improved cancer-related fatigue recovery through acupuncture at LI4, KI3, ST36, and SP6 [43]. The use of laser acupuncture at HT7, PC6, KI3, and SP6 for insomnia resulted in improved sleep latency, fewer nighttime awakenings, and increased sleep efficiency [21]. However, this study supports the therapeutic effect of stimulating acupoints along the meridians, which are believed to influence the mechanisms of acupuncture therapy that regulate physiological functions [44], in treating post-dialysis fatigue. Notably, this study found that fatigue was continuously associated with both dialysis and depressive symptoms, while sleep quality was linked continuously to dialysis symptoms. This supports previous studies, which excluded severe depression or mental illness, showing that acupressure alleviated fatigue in HD patients [41, 45]. More importantly, this study aimed to highlight how dialysis and depressive symptoms continually influence fatigue and sleep quality in patients undergoing HD.

Three patients in this study experienced transient, mild tingling sensations and minor petechiae possibly related to lupus erythematosus [46]. However, a systematic review of randomized controlled trials found that laser acupuncture was safe in 14 trials, reporting no adverse effects. In contrast, six other trials observed mild symptoms such as tingling, pain flare-ups, and transient fatigue; these symptoms generally resolved on their own within a day [47]. Another systematic review reported that acupuncture caused pain, headache, fatigue, and tenderness, though these adverse events were seen as acceptable [27]. This study suggests that laser acupuncture is a safer and less painful treatment compared to traditional methods of acupuncture.

This study conducted a double-blind, randomized, placebo-controlled trial; however, it has some limitations. First, recruiting participants from only one medical center may limit the ability to generalize the results. Second, this study only examined the short-term effects of laser acupuncture over a six-week period. The observed improvements do not necessarily reflect long-term improvements in physical health or sleep quality. Finally, although the study controlled for the effects of dialysis and depressive symptoms through research design and statistical analysis, it relied on subjective questionnaires to assess outcomes. It did not include physiological measures such as biomarkers or blood concentrations.

## Conclusion

This double-blind, randomized, placebo-controlled trial provides evidence that six weeks of laser acupuncture benefits patients with ESRD undergoing HD by alleviating post-dialysis fatigue, reducing recovery time, and improving sleep quality, all while excluding the continuous influences of dialysis and depressive symptoms. Additionally, because of its non-invasive nature, safety, and short treatment duration, laser acupuncture is suggested as a treatment option in clinical practice. It can be applied at seven specific acupoints: LI4, HT7, PC6, KI3, ST36, GB34, and SP6, starting from the second hour of the HD procedure. For the best results, laser acupuncture should be performed for at least two weeks. Future studies should investigate the long-term effects of laser acupuncture on patients undergoing HD. This study suggests that involving multiple centers could strengthen its significant findings.

## Supplementary Information

Supplementary Material 1

## Data Availability

The data that support the findings of this study are available from the corresponding author upon reasonable request.

## Abbreviations

BDI: Beck Depression Inventory
DSI: Dialysis Symptom Index
ESRD: End-stage renal disease
GB34: Yanglingquan
HD: Hemodialysis
HT7: Shenmen
KI1: Yongquan
KI3: Taixi
LA: Laser acupuncture
LI4: Hegu
PC6: Neiguan
PSQI: Pittsburgh Sleep Quality Index
RN6: Qihai
SP6: Sanyinjiao
ST36: Zusanli
